# Prevalence of sickle cell trait and its association to renal dysfunction among blood donors at University of Medical Sciences Teaching Hospital, Ondo, Nigeria

**DOI:** 10.4314/ahs.v21i3.33

**Published:** 2021-09

**Authors:** Akinwumi Ayodeji Akinbodewa, Adeyemi Ogunleye, Oluseyi Ademola Adejumo

**Affiliations:** 1 Kidney Care Centre, department of Medicine, University of Medical Sciences Teaching Hospital, Ondo State; 2 Department of Medical Laboratory Services, University of Medical Sciences Teaching Hospital, Ondo State

**Keywords:** Haemoglobin genotype, sickle cell trait, renal function, blood donor, Nigeria

## Abstract

**Introduction:**

Prospective blood donors are routinely screened for blood borne infections but medical illnesses and haemoglobin genotype are overlooked despite a high prevalence of haemoglobin AS among Nigerian donors.

**Objective:**

To determine the prevalence of haemoglobin AS and its association to renal function, if any.

**Method:**

Apparently healthy donors were studied between February and December 2018. Their haemoglobin genotype and, estimated glomerular filtration rates were determined.

**Results:**

There were 96 males (94.1%) and 6 (5.9%) females with mean age of 26.7±4.5 years (range 19–44 years) and mean eGFR of 103.97±19.00ml/min/1.73m2. Eighty one (79.4%) and 21 (20.6%) subjects had haemoglobin AA and AS genotypes respectively. The mean eGFR for subjects with haemoglobin AA and AS were 105.2±18.6ml/min/1.73m^2^ and 99.9 ± 21.2ml/min/1.73m^2^ respectively (p value = 0.270). Eighty one (79.4%), 20 (19.6%) and 1 (1.0%) subjects had renal function at >90ml/min/1.73m^2^, 60–89ml/min/1.73m^2^ and 30–59ml/min/m^2^ respectively. There was no significant difference in the mean eGFR between subjects with haemoglobin AA and AS (mean difference 5.3, p = 0.265, 95%CI = -4.07 to 14.60).

**Conclusion:**

The prevalence of sickle cell trait among Nigerian blood donors is high. There is no significant difference in the renal function status of blood donors with SCT and normal haemoglobin genotype.

## Introduction

Blood transfusion, by all standards should be a safe, easy and fast life-saving procedure. It should be conducted in a manner that the health of recipients and donors alike is protected. The World Health Organization (WHO) guidelines on assessing donor suitability provides clinicians and blood bank personnel with protocols for acceptance and/or deferment of blood donors with sickle cell trait (SCT) and those with chronic kidney disease (CKD).^1^ However, blood banking practice in most hospitals is often limited to screening of prospective donors for certain communicable diseases such as Human Immunodeficiency Virus (HIV), hepatitis B and C viruses, syphilis and other relevant transmissible diseases with little emphasis on medical background check.^2^–^3^

This approach often times gives an impression that the focus of the laboratory personnel is directed more at protecting would-be recipients without taking into consideration the adverse effects of blood donation on prospective donors, especially those who may have background medical illnesses. For instance, in one large study, a significant number of donors had measurable untoward systemic adverse effects such as fatigue, vasovagal symptoms, nausea and vomiting after donation for blood transfusion.^4^

Current studies indicate that the prevalence of haemoglobin S among blood donors in Nigeria is high, ranging between 19.8% and 26.1%.^5^–^6^ A combination of factors have contributed to the high presence of people with SCT in the donor pool in Nigeria.^2^,^7^–^10^ Firstly, in most hospitals, people with SCT are not routinely screened and deferred from blood donation (where necessary) due to pressure of demand on blood bank units for pints of blood.^7^ Secondly, due to economic recession and need for pecuniary gains in order to make ends meet, HbS carriers have been found among donors, especially in the commercial group.^8^–^10^ Related to this is the higher number of commercial donors over voluntary non-remunerable donors, a trend which presupposes lesser adherence to ethics and protocols. Indeed, a survey by the National Blood Transfusion Service in Nigeria showed that commercial donors form 25% of donors in public hospitals and 75% in the private sector.^2^

There are indications for physicians to pay closer attention to the haemoglobin genotype of would-be blood donors as there are new evidences suggesting that individuals with SCT are prone to developing chronic kidney disease (CKD).^11^–^13^ Added to this is the documented evidence that individuals with SCT (AS haemoglobin genotype) can develop ill-health from blood donation. For example, available data indicates that individuals with the SCT who are prone to vaso-occlusive crisis (surgery, prolonged hypoxaemia, pre-existing vaso-occlusive disease) should be excluded from blood donation.^14^

Other established indications for excluding such people from blood donation include neonatal transfusion, exchange blood transfusion, leukofiltration and in cases where blood transfusion is required for a sickle cell anaemia patient.^2^, ^15^ This leaves room to advocate for routine haemoglobin genotype screening for prospective blood donors and risk assessment of those with haemoglobin AS for renal dysfunction. We therefore set out to determine the pattern of distribution of haemoglobin genotype among blood donors in a tertiary hospital in Nigeria, and their association to renal dysfunction, if any.

## Method

This was a descriptive, cross-sectional study conducted at the blood bank unit of the kidney care centre, University of Medical Sciences Teaching Hospital, Ondo State, Southwest Nigeria from 5^th^ February 2018 to 31st December 2018.

### Inclusion criteria

A representative sample of one hundred and two consecutive, apparently healthy individuals aged between 19 and 44 years who came to donate blood were selected by convenience sampling method.

A minimum sample size of 74 for this study was calculated from the prevalence of 5.1% obtained for renal dysfunction among young adults with SCT.^13^ However due to possibility of attrition, the number was increased to 110.

The formula, z2pq/d^2^ for sample size determination in study population less than 1,000 subjects was used^16^, where;

p = proportion in the target population estimated to have a particular characteristics

z = the standard normal deviation (using 95% confidence level = 1.96)

d = degree of accuracy desired, set at 0.05

q= 1.0 – p

### Exclusion criteria

all pregnant and lactating women, persons younger than 18 years or older than 60 years and persons with the following: haemoglobin concentration < 13.5g/dl for males and 12.5g/dl for females, positive serology testing for Human Immunodeficinecy Virus I and II (HIV I and II), hepatitis B surface antigen (HBsAg) and anti-hepatitis C virus (Anti-HCV), persons who appear generally unwell, breathless, or having an active fever, cough or undergoing treatment for a medical illness, persons who donated within the last 12 weeks for males and 16 weeks for females were excluded from the study.^1^

Blood samples were obtained for blood group, haemoglobin genotype and serum creatinine. Venous blood sample for analysis was collected at the media-cubital fossa from each participant. Two milliliters (ml) of blood was dispensed into the ethylene diamine tetra-acetate (EDTA) blood collection tubes for determination of haemoglobin genotype using the standard haemoglobin electrophoresis machine (alkaline medium, MICROFIELD, England). Assessment of serum creatinine was performed using 3 ml of venous blood dispensed in lithium heparin collection tubes. Samples were centrifuged in the laboratory to separate the serum which was then analysed by the modified kinetic Jaffe method using the RANDOX, UK commercially prepared reagent.

The estimated glomerular filtration rate (eGFR) was determined using the chronic kidney disease-epidemiology (CKD-EPI) calculator. The CKD-EPI was developed from a large database of people, including those with and without kidney disease, diabetes, and a history of organ transplantation and it takes into consideration gender in the estimation of eGFR.^17^

### Data analysis

Data was analyzed using SPSS version 20. Continuous variables were presented as mean ± standard deviation for un-skewed data. Discrete variables were presented as frequency and percentage. Comparison of means was carried out using the independent samples T test. P value < 0.05 was taken to be significant at 95% Confidence Interval.

### Ethical approval

Ethical clearance for this research was sought from the Ethics and Research Committee of State Specialist Hospital, Akure, Ondo State. All the participants gave their informed consent before their samples were collected.

## Results

The participants consisted of 96 males (94.1%) and 6 (5.9%) females with mean age of 26.7±4.5 years (age range 19–44 years) and mean serum creatinine value of 100.5umol/L. Seventy three (71.6%), 15 (14.7%) and 9 (8.8%) had O Rhesus D positive, A Rhesus D positive and B Rhesus D positive blood groups respectively (table 1). Sixty six (64.7%), 34 (33.3%) and 2 (2.0%) had packed cell volume within the ranges of 36–40%, 41–45% and >45% respectively (table 1) Eighty one (79.4%), 20 (19.6%) and 1 (1.0%) subjects had renal function at >90ml/min/1.73m^2^, 60–89ml/min/1.73m^2^ and 30–59ml/min/m^2^ respectively (table 1). There was no subject with renal function lower than 30ml/min/1.73m^2^ in this study.

**Table 1:** Demographic and clinical parameters of participants

Parameters	Frequency (N)	Percentage (%)
Gender		
Male	96	94.1%
Female	6	5.9%
Age group (years)		
19–24	36	35.3%
25–29	40	39.2%
30–34	20	19.6%
>34	6	5.9%
Haemoglobin genotype		
AA	77	75.5%
AC	4	3.9%
AS	21	20.6%
Blood group		
A Rhesus positive	15	14.7%
A Rhesus negative	1	1.0%
B Rhesus positive	9	8.8%
B Rhesus negative	1	1.0%
AB Rhesus positive	0	0
AB Rhesus negative	0	0
O Rhesus positive	73	71.6%
O Rhesus negative	3	2.9%
Renal function classification		
(ml/min/1.73m2)		
>90	81	79.4%
69–89	20	19.6%
30–59	1	1.0%

Seventy seven (75.5%), 4 (3.9%) and 21 (20.6%) subjects had haemoglobin AA, AC and AS genotypes respectively and, the proportion of females with haemoglobin AS was higher than males (table 2).

**Table 2:** Gender distribution of haemoglobin genotype among participants

Gender	Haemoglobin genotype			Total
	AA	AC	AS	
Male	74 (77.1%)	4 (4.2%)	18 (18.7%)	96 (100.0%)
Female	3 (50.0%)	0 (0.0%)	3 (50.0%)	6 (100.0%)
Total	77 (75.5%)	4 (3.9%)	21 (20.6%)	102 (100.0%)

The mean packed cell volume and mean eGFR were 103.97±19.00ml/min/1.73m^2^ and 40.4±2.3% (range, 36–49%) respectively. The mean eGFR for subjects with haemoglobin AA, AC and AS were 105.2±18.6ml/min/1.73m^2^, 102.6 ± 16.1 ml/min/1.73m^2^ and 99.9±21.2ml/min/1.73m^2^ respectively (table 3). There was no significant difference in the mean eGFR between subjects with haemoglobin AA and AS (mean difference 5.3, p = 0.265, 95%CI = -4.07 to 14.60).

**Table 3:** Association between haemoglobin genotype and renal function

Haemoglobin genotype	eGFR (mean ± SD)	Mean difference	Standard Error difference	P value	95% Confidence Interval
AA	105.0±18.4	5.157	4.648	0.270	-4.064 to 14.3778
AS	99.9±21.2				
	PCV				
AA	(mean ± SD) 40.3±2.1	-0.788	0.568	0.169	-1.916 to 0.340
AS	41.1±2.9				

P is significant at value < 0.05

## Discussion

Majority (94.1%) of the study participants were males which is similar to reports from within and outside Nigeria. 18–20 This has been attributed to the use of more stringent rules which do not very much encourage prospective female blood donors which include but not limited to on-going menstruation, pregnancy and lactation. 21 Socio-culturally, women are not also generally encouraged to donate due to perceived weaker bodies.^19^,^22^

The prevalence of blood donors with SCT (21.6%) was high in our study. This figure falls within the range already established from earlier studies in Nigeria.^5^–^6^ It is higher than 11.3% reported in a Ghanaian study in neighbouring West Africa.^23^ This is probably associated to the already high prevalence of SCT in the general population in Nigeria; an approximated one-quarter of Nigerians are known to be carriers of the haemoglobin S gene (SCT).^13^,^24^–^25^ It therefore goes without saying, that individuals with SCT would have been lumped into the general blood donor pool unknowingly from the large population of people with SCT. Indeed, it has been shown from earlier studies that only a minute fraction of donors, including those with SCT are aware of their sickle cell status and haemoglobin genotypes.^23^,^26^

In 2018, the Federal Government of Nigeria approved an Executive bill for the establishment of a National Blood Service Commission but this is still awaiting passage into law.^27^–^29^ This delay has encouraged unethical practices in the blood transfusion units with individuals who have haemoglobin levels below the acceptable cut-off participating in blood donation, even though this is more common in the private sector where pecuniary gain is the main motive for blood donation. For the same reason, some commercial donors are known to hide their blood donation history, especially those who have done so more frequently than expected.^30^ It is therefore possible that a sizeable number of haemoglobin AS donors with low haemoglobin concentration would have been bled with potential damage to nephrons owing to anaemic hypoxaemia. For instance, Benedict et al demonstrated a lower haemoglobin concentration among paid donors compared to voluntary donors (7.7±2.9 vs 13.9±1.2, P < 0.001).^31^ These further underscore the need for the bill to be passed into law as quickly as possible. Also, the appropriate regulatory body must develop a structure that will ensure its enforcement across the nation.

It has been postulated that SCT may directly lead to loss of renal function through episodic sickling leading to chronic parenchymal ischaemia. Low oxygen content of the renal medulla may provide an enabling setting for intravascular sickling which may lead to chronic microvascular damage and, consequently to chronic hypoxia and tubule-interstitial fibrosis.^32^

A little over one-fifth of the subjects in our study had renal function below optimum (90ml/min/1.73m^2^) while 1% had renal function below 60ml/min/1.73m^2^. The relatively lower mean eGFR among donors with SCT even though not to the significant level (mean difference 5.3, p = 0.265, 95%CI = -4.07 to 14.60) suggests that there may be an association between SCT and CKD. It is possible that with a larger number of subjects, the mean difference may become significant. This finding suggests that there may be a larger number of individuals with the SCT who have concomitant CKD who are in the donor pool who require screening, detection and exclusion from blood donation. This process need not be limited to haemoglobin genotype but extended to cover other medical illnesses such as hypertension, diabetes, dyslipidaemia etc. For instance, there are reports indicating that co-existence of SCT with other chronic medical diseases (such as diabetes mellitus and autosomal polycystic kidney disease) is associated with higher risk of developing CKD.^33^–^34^

## Limitations

This was a cross-sectional study with the data skewed towards the male gender. Confounding variables such as hypertension, diabetes, obesity, smoking and other cardiovascular risk factors for chronic kidney disease were not excluded at the blood transfusion unit.

## Conclusion

The prevalence of SCT among Nigerian blood donors is high and there is no significant difference in the renal function status of blood donors with normal haemoglobin and those with the SCT. There is a need to conduct high powered, larger prospective studies to improve the reliability of outcome when determining relationship between SCT and renal dysfunction among blood donors.
